# Multi-Gait In-Pipe Locomotion via Programmable Friction Reorientation

**DOI:** 10.3390/biomimetics11040285

**Published:** 2026-04-20

**Authors:** Jaehyun Lee, Jongwoo Kim

**Affiliations:** Biomedical and Intelligent Robotics Laboratory, Department of Mechanical Engineering, Kyung Hee University, 1732 Deogyeong-daero, Yongin 17104, Republic of Korea; 2019100726@khu.ac.kr

**Keywords:** continuum robots, programmable friction, multimodal locomotion, biologically-inspired robots, in-pipe inspection, minimal actuation, soft robotics

## Abstract

In-pipe robots must navigate narrow, curved passages where rigid mechanisms often require bulky steering units. Soft crawlers offer better compliance but typically rely on multiple actuators or reconfigurable contacts to achieve multi-directional motion. Drawing inspiration from biological soft crawlers that exploit directional friction and coordinated anchor–slip patterns, this study focuses on locomotion principles observed in caterpillars, water boatmen, and whirligig beetles. Based on these bioinspired concepts, we present a tendon-driven soft in-pipe robot that combines continuum bending–twisting deformation with modular anisotropic friction pads (AFPs), enabling three locomotion modes using only two motors. AFP inclination, curvature, and ridge geometry were optimized through friction tests, constant-curvature modeling, and finite element analysis to enhance directional adhesion on flat and curved surfaces. A deformation-based locomotion framework was developed to couple tendon actuation with friction orientation, achieving longitudinal crawling, transverse translation, in-place rotation, and smooth transitions via programmed twisting. Driving experiments demonstrated repeatable anchor–slip locomotion with average speeds of 28.6 mm/s, 15.7 mm/s, and 11.5°/s for the three modes. Pipe tests in straight, curved, and T-junction sections further validated stable contact and reliable gait transitions. These findings highlight the potential of friction-programmed continuum robots as compact, bioinspired platforms for advanced in-pipe inspection and diagnostic tasks.

## 1. Introduction

Soft crawling robots refer to a class of soft mobile robots that generate locomotion through periodic body deformation, friction modulation, and distributed compliance along the body [1,2,3,4,5,6,7]. Various actuation mechanisms, such as pneumatic and hydraulic chambers, artificial muscles, electrohydraulic actuators, thermal actuators, and tendon-driven bending modules, have been integrated into film-based bodies, modular segments, metamaterial skins, and interlocking architectures to realize diverse crawling patterns across different environments [4,5,8,9,10,11,12]. In contrast to rigid crawling robots, which rely on link–joint structures and often suffer from contact instability, jamming, and poor environmental adaptability in confined or cluttered spaces, soft crawling robots exploit continuum deformation to maintain body–surface conformity, distribute contact forces, and dissipate collisions effectively [1,7,13,14,15,16]. Film-type and modular soft crawlers have demonstrated stable locomotion over rough terrains, inclined surfaces, underwater substrates, and irregular boundaries [8,17,18,19], while wheel–leg hybrids and interlocking pneumatic architectures have been proposed to extend the range of attainable locomotion modes beyond those of conventional rigid platforms [9,20,21]. Among these implementations, structurally simple continuum mechanisms based on origami-inspired patterns or tendon-driven backbones have drawn increasing attention, as they can generate rich combinations of bending and twisting deformations without requiring complex joint assemblies [22,23,24]. Such structural simplicity offers useful design principles for soft crawling platforms intended to operate in constrained environments.

Soft crawling robots have been extensively inspired by biological organisms such as earthworms, caterpillars, larvae, and snakes. Prior work has categorized their locomotion strategies into peristaltic waves, inchworm-like anchoring, rectilinear and lateral undulation, buckling-based snapping, and unidirectional propulsion enabled by frictional anisotropy [1,2,4,25]. Pneumatic multi-chamber earthworm robots generate peristaltic locomotion through phase-shifted inflation and deflation [17,26,27], while multistable ribbon structures and origami-based metamaterial actuators leverage buckling instabilities to achieve propulsion inside tubes and other confined spaces [28,29]. Thermally actuated caterpillar-like crawlers and electrohydraulic modular structures further demonstrate rapid forward and backward crawling through sequential bending and anchoring [4,9,10,11]. A common feature across these biological and bioinspired systems is that they generate diverse locomotion patterns not by relying on highly articulated skeletal structures, but by coordinating local body bending and segment interactions to modulate frictional contacts. The in-pipe soft crawling robot proposed in this study is likewise inspired by biological examples such as caterpillars, water boatman insects, and whirligig beetles, and aims to distill their coordination between body deformation and frictional coupling into an engineering principle for in-pipe locomotion.

At the material and surface levels, various anisotropic friction structures have been developed using magnetorheological elastomer pads, micro-patterned skins, and directional fibers, enabling passive unidirectional motion and enhanced anchoring [19,30,31,32]. However, most existing systems remain focused primarily on axial motion, and architectures based on multiple chambers and multiple actuators often incur complexity in pneumatic routing, control channels, and synchronization, which hampers their deployment in confined and mechanically constrained environments [2,4,5,25,33,34]. Some studies have demonstrated a limited set of distinct modes, such as switching between forward and backward crawling or between crawling and inchworm-like anchoring [10,11,17,20,21], yet an integrated design principle for generating and continuously transitioning among multiple gaits within a single soft crawling platform is still largely missing [1,4,25]. In particular, anisotropic friction structures are often treated as static anchoring elements rather than as components of a reconfigurable frictional landscape that can be actively repositioned through deformation of a continuum body.

Pipelines represent one of the most geometrically constrained environments for mobile robots, involving variations in curvature and diameter, elbows and T-junctions, weld beads, deposits, and local surface irregularities [16,35,36,37]. Rigid in-pipe robots that rely on wheels, clamps, or rigid backbones struggle to maintain stable contact in such environments and are prone to collisions, jamming, and loss of traction [16,35,36]. Continuum-based soft in-pipe robots, on the other hand, can conform to inner-wall curvature and maintain distributed contact, offering advantages in navigating regions with changing curvature and diameter [28,29,34,38]. Nevertheless, most soft in-pipe robots are still limited to axial locomotion, and only a few provide any form of lateral translation or continuous in-place rotation [26,28,29,34,38,39]. Moreover, simple rubber pad-based anchoring structures tend to exhibit unstable traction when interacting with welds, gaps, or surface irregularities along the pipe interior [16,36,37].

A conceptual comparison with representative soft crawling and in-pipe robots is summarized in Table 1, including representative systems reported by Kim et al. [40], Lin et al. [39], Yeh et al. [34], and Zhao et al. [41]. This comparison highlights that most prior systems are limited to axial locomotion and rely on passive friction asymmetry or anchoring mechanisms, whereas the present work uses deformation-induced friction reorientation to expand the locomotion repertoire.

Recent reviews have highlighted the need for new soft in-pipe platforms that combine structural simplicity with multi-directional mobility [4,15,16,37]. From this perspective, the in-pipe environment provides a compelling testbed for integrating continuum deformation mechanisms with anisotropic friction design, in order to realize multiple gaits using a small number of actuators.

In this study, we propose a tendon-driven soft in-pipe crawling robot that combines continuum bending and twisting with modular anisotropic friction pads (AFPs). The robot’s body consists of a tendon-driven continuum backbone capable of generating coordinated combinations of bending and twisting, and its outer surface is equipped with AFPs whose frictional axes are oriented differently around the circumference. The relationship between body deformation and friction orientation is analogous to how a caterpillar bends its body segments to shift the location of ground contact and how a water boatman adjusts the angle and sweep of its paddling legs to generate directional thrust, while also drawing inspiration from the rapid pivoting maneuvers observed in whirligig beetles. Building on this bioinspired perspective, the key idea of the proposed platform is to use continuum twisting and bending, rather than mechanical switches or multiple actuators, to actively reorient the AFPs with respect to the environment. This deformation-induced friction reorientation enables the robot to generate three distinct gaits—longitudinal (caterpillar-like) crawling, transverse (water-boatman-like) lateral translation, and rotational (whirligig-beetle-like) in-place turning—with only two driving motors. Unlike prior soft crawling robots that primarily demonstrate axial crawling, the proposed system extends the achievable motion repertoire to axial forward/backward crawling, transverse translation, and in-place rotation using only two actuators. In this sense, the proposed robot can be viewed as an engineering simplification of biological neuromuscular coordination, compressing rich multi-gait behavior into coordinated control of a minimal set of actuators.

The AFPs employed in this work are designed as detachable and modular units whose curvature radius, base inclination angle, and ridge geometry can be tailored to different substrate and pipe conditions. This modularity allows the frictional properties of the robot to be rapidly reconfigured for different environments without altering the continuum backbone or the actuation hardware. The design is informed by prior work on programmable anisotropic friction, where out-of-plane deformation and surface patterning are used to encode direction-dependent frictional responses [40]. Here, we extend this concept by embedding such programmable friction structures into an in-pipe crawling platform and explicitly coupling them with tendon-driven continuum deformation to achieve multi-gait locomotion.

To analyze how combinations of bending and twisting deformations reposition the AFPs and induce transitions among different gaits, we develop a deformation-based modeling framework that links tendon actuation, backbone kinematics, and frictional contact states. Finally, we experimentally demonstrate that the proposed robot can achieve robust longitudinal crawling, lateral translation, and in-place rotation on flat stainless-steel substrates and inside curved pipe sections. These results suggest a design principle for soft in-pipe crawling platforms that combine structural simplicity, multi-directional mobility, and environmental adaptability through deformation-induced friction reorientation.

The remainder of this paper is organized as follows. Section 2 describes the overall robot architecture, tendon actuation system, and the design and fabrication of the frictional structures. Section 3 formulates the deformation-based locomotion principles and explains the mechanism by which transitions between movement modes occur. Section 4 presents experimental validations and quantitative locomotion performance across various pipe environments. Section 5 discusses the implications, advantages, and potential extensions of the proposed approach. Finally, Section 6 concludes the paper.

## 2. Design and Fabrication

### 2.1. Robot Design and Actuation

**Figure 1:**
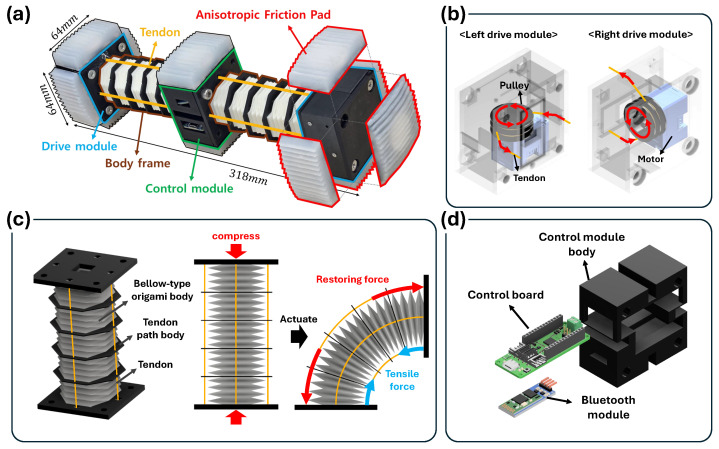
Structural configuration of the proposed tendon-driven in-pipe crawling robot. (**a**) Overall configuration of the proposed tendon-driven in-pipe crawling robot. (**b**) Drive module assembly including the motor–pulley system and the tendon-driven actuation mechanism. (**c**) Bellow-type origami body frame and its deformation behavior under tendon-induced forces. (**d**) Control module containing the motor control board and Bluetooth module for remote control.

The overall configuration of the proposed tendon-driven in-pipe crawling robot, shown in Figure 1a, consists of two drive modules, a central control module, and an origami-based body frame. A drive module is installed at each end of the robot, while the central section contains a body frame that provides structural compliance. The control module is positioned at the center of the robot and integrates the electronic components required for actuation and communication. Four tendons—upper, lower, left, and right—are routed along the body frame and connected to the drive modules at both ends. At each end of the robot, an Anisotropic Friction Pad (AFP) that enables multi-gait locomotion is attached using 3M Dual Lock fasteners (3M, St. Paul, MN, USA) for easy replacement. Excluding the AFPs, the robot’s body measures 64 mm × 64 mm × 318 mm and weighs 200 g.

As illustrated in Figure 1b, each drive module contains a motor coupled with a pulley around which two tendons are pre-wound—one clockwise and the other counterclockwise. When the motor rotates clockwise, the counterclockwise-wound tendon is pulled while the clockwise-wound tendon is released. The distal ends of these tendons are anchored to the opposite drive module, so a single motor rotation produces opposite length changes in the tendon pair. In this configuration, the motor of one drive module actuates the left and right tendons, whereas the motor in the opposite module is oriented 90° apart and actuates the upper and lower tendons. For tendon actuation, a Dyneema braided line (0.18 mm diameter) was used due to its high tensile strength and low elongation. Each drive module is actuated by a Dynamixel XC330-M288-T servo motor (ROBOTIS, Seoul, South Korea) configured in the extended position mode, which provides multi-turn position control with an encoder resolution of 4096 counts per revolution. The motor operates using its built-in PID controller (P = 1800, I = 0, D = 200) with a profile velocity of 1500 and a profile acceleration of 30, ensuring smooth and repeatable tendon winding during operation. All tendon actuation was commanded through relative goal-position updates referenced to the current encoder value, enabling precise and consistent control of tendon length changes required for the robot’s locomotion mechanisms.

The body frame, shown in Figure 1c, is fabricated through 3D printing and designed as a bellow-type origami structure that provides both flexibility and elastic recovery. To ensure sufficient resilience and fatigue resistance during repeated bending and twisting, the frame was printed using NinjaFlex (NinjaTek, Manheim, PA, USA) (flexible TPU filament). NinjaFlex is a thermoplastic polyurethane widely used in soft robotic structures due to its high elasticity and durability under repeated deformation, with an elongation at break of approximately 660% according to the manufacturer’s material specification. This geometry offers high torsional stiffness, enabling the robot to bend effectively without undesired twisting during tendon-driven actuation. A rigid tendon-path body is embedded between the origami folds to guide the tendons along predetermined routes. The frame is maintained in a compressed configuration by the tendon pre-tension, increasing the restoring energy around the neutral position. When one tendon is wound, the tendon-induced tensile force pulls that side of the body while the opposite side extends due to the origami structure’s elastic recovery, resulting in a bending deformation.

The central control module, depicted in Figure 1d, houses the motor control board and a Bluetooth communication module. An HC-06 Bluetooth module (generic module, Guangzhou, China) was used to enable wireless control commands. All wiring between the motors and the control board is routed internally through the body frame to prevent external exposure. Although the robot receives power through a wired connection, control signals are transmitted wirelessly via the Bluetooth module.

### 2.2. Friction Pad Design

**Figure 2:**
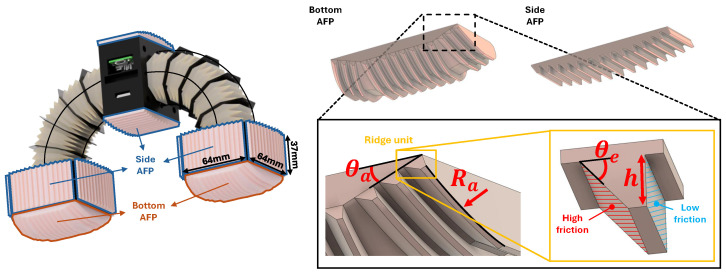
Configuration and geometric design of the AFPs. Demonstration of the bottom and side AFPs mounted on the robot, including their geometric parameters ($\theta_{a}$, $R_{a}$, $\theta_{e}$, *h*) and the anisotropic friction properties that enable diverse locomotions.

The overall configuration of the robot during crawling is shown in the left panel of Figure 2. AFPs are mounted according to the geometry of each module located at both ends of the robot. The AFP attached to the bottom of the drive module is defined as the bottom AFP, whereas those attached to the side surfaces of the drive module and the control module are defined as the side AFPs. This layout assigns different locomotion roles to each AFP, in combination with anisotropic friction, enabling longitudinal, transverse, and rotational movements.

The AFP geometry is determined by two design parameters: $\theta_{a}$, which ensures a smooth and continuous ground contact during longitudinal crawling, and $R_{a}$, which enables adaptive conformity to curved pipe surfaces. The bottom AFP uses $\theta_{a}$ to maintain stable and continuous ground contact throughout the crawling cycle, whereas the side AFPs rely solely on $R_{a}$ to conform stably to the curved inner wall. Thus, each AFP is functionally specialized to support its corresponding locomotion mode.

Multiple ridge units are arranged at the distal ends of the AFPs. Each ridge unit is characterized by two geometric parameters—the inclination angle $\theta_{e}$ and the height *h*—which govern the intrinsic frictional characteristics of the silicone surface. These parameters were examined in a parametric study to identify the configuration that yields the highest friction. Anisotropic friction is then achieved by selectively applying low-friction coatings to specific ridge surfaces, creating a directional frictional asymmetry. Because each AFP consists of multiple ridge units aligned with the same frictional orientation, the AFP as a whole functions as a directionally asymmetric friction surface. This AFP is directly utilized in the locomotion mechanisms described in Section 3.

#### 2.2.1. Pad Fabrication

**Figure 3:**
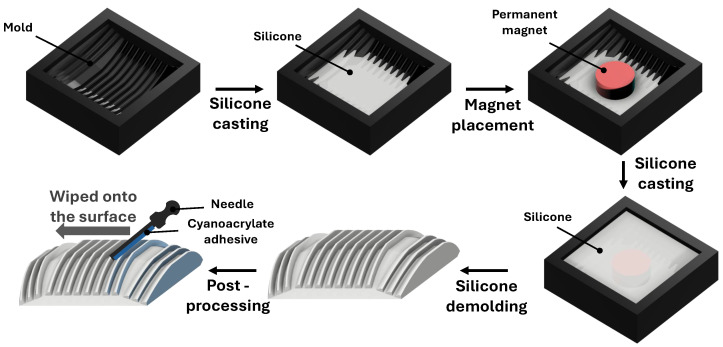
Fabrication process of the silicone AFP with magnetic and anisotropic friction properties. The process includes silicone molding, magnet embedding, and selective thin-film coating, which together form the ridge geometry and impart anisotropic friction to the AFP.

The step-by-step fabrication and post-processing procedure of the silicone ridge unit is illustrated in Figure 3. First, a mold with the ridge geometry was fabricated using 3D printing, and a predetermined amount of Ecoflex™ 00-10 silicone (Smooth-On, Macungie, PA, USA) was poured to perform the first casting. Once the silicone was partially cured, a permanent magnet was placed at the center of the upper surface to enhance adhesion with the metallic pipe. A TEF-D0100 neodymium disk magnet (Webcraft GmbH, Gottmadingen, Germany) was used for this embedding step to provide strong magnetic attraction while maintaining compact size. Subsequently, an additional amount of silicone was poured for the second casting process. After complete curing, the structure was demolded, resulting in a silicone body with the designed ridge geometry.

Next, a post-processing step was conducted to modify the anisotropic friction properties of the ridge unit. A thin layer of cyanoacrylate adhesive was applied onto the specific ridge surfaces where friction reduction was desired and left to dry naturally, forming a thin surface film. This film possesses a lower friction coefficient than the underlying silicone. When a coated ridge makes contact with the ground, the thin film becomes the actual interface interacting with the surface, thereby producing the intended low-friction behavior. Because the film is sufficiently thin, it conforms to the deformation of the compliant silicone body without noticeably altering its elasticity or overall mechanical response.

By coating only selected ridge surfaces, friction is reduced exclusively in the targeted regions, while uncoated areas fully retain the high-friction characteristics of bare silicone. This localized surface-treatment strategy allows precise shaping of anisotropic friction within the AFP structure and ensures that regions with different frictional roles operate without interfering with one another.

#### 2.2.2. Frictional Characterization of Ridge-Unit Geometries

**Figure 4:**
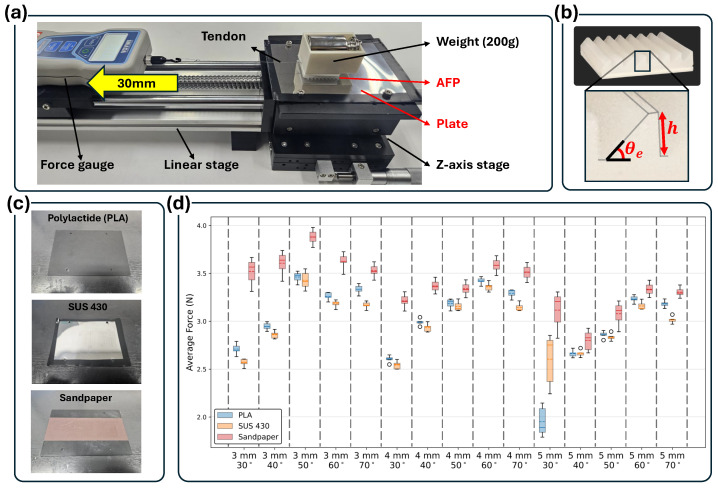
Experimental setup and friction test results for the ridge units. (**a**) Friction-test setup using a linear stage and force gauge. (**b**) Geometric parameters of the ridge unit ($\theta_{e}$ and *h*). (**c**) Test plates made of PLA, SUS 430, and sandpaper. (**d**) Measured friction forces for all combinations of $\theta_{e}$ and *h* on each plate type.

The experimental setup used to evaluate the frictional characteristics of the ridge units is presented in Figure 4. The setup consists of a force gauge mounted on a linear stage, where the test plate is fixed to the stage and the AFP specimen is placed on top of it. A controlled horizontal displacement of 30 mm was applied at a constant speed of 5 mm/s, while a normal load of 200 g was applied vertically to replicate realistic contact conditions during locomotion.

The geometric parameters of the ridge unit, characterized by the inclination angle $\theta_{e}$ and ridge height *h*, are illustrated in Figure 4b. These two parameters determine the baseline frictional behavior of the silicone surface.

The three types of test plates used in the experiment—Polylactide (PLA), SUS 430, and sandpaper—are shown in Figure 4c. For the sandpaper surface, a DEERFOS KA162 abrasive paper (320 grit) (DEERFOS Co., Ltd., Incheon, South Korea) sheet was used to provide a high-friction abrasive condition suitable for evaluating the ridge geometry under rough-contact environments. Among the three plates, the surface friction level increases in the order of SUS 430, PLA, and sandpaper, providing low-, medium-, and high-friction contact conditions for evaluating the ridge units.

The measured friction forces for all combinations of inclination angle ($\theta_{e}=30^{{}^{\circ}},$$40^{{}^{\circ}},50^{{}^{\circ}},60^{{}^{\circ}},70^{{}^{\circ}}$) and ridge height (h=3,4,5mm) are summarized in Figure 4d. Each condition was tested five times in randomized order for statistical reliability. The box plots represent the median and interquartile range (IQR), and the whiskers extend up to 2.0× IQR, with values beyond this range treated as outliers. Among all configurations, the ridge with h=3mm and $\theta_{e}=50^{{}^{\circ}}$ exhibited the highest friction across all plate types. Excessively large ridge heights reduced the effective contact area, while small inclination angles generated insufficient shear stress, explaining the observed trends.

These results confirm that geometric optimization of the ridge structure plays a critical role in enhancing the intrinsic frictional behavior of silicone. The identified optimal configuration also provides important design guidance for achieving anisotropic friction characteristics, discussed in the following section.

#### 2.2.3. Durability Test of Anisotropic Friction Pad

To evaluate the durability of the anisotropic friction interface under repeated sliding conditions, a cyclic reciprocating friction test was conducted. Since the anisotropic friction of the AFP is generated by selective cyanoacrylate thin-film coating, it is necessary to verify that the directional friction characteristics remain stable during long-term operation.

**Figure 5:**
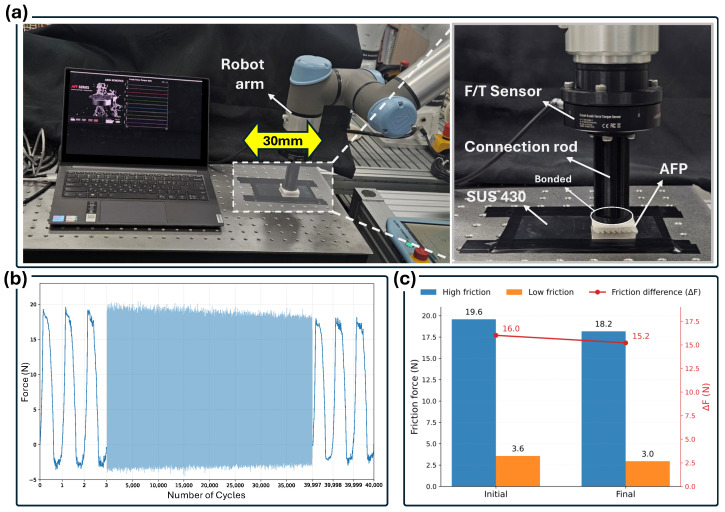
Durability test of the anisotropic friction pad (AFP) under repeated sliding cycles. (**a**) Experimental setup using a robotic arm, F/T sensor, and bonded AFP sliding on a SUS 430 plate with a reciprocating stroke of 30 mm. (**b**) Friction force history measured during 40,000 repeated sliding cycles. (**c**) Comparison of high-friction and low-friction forces and the corresponding friction difference (ΔF) between the initial and final cycles.

As shown in Figure 5, the AFP specimen was attached to a connection rod connected to a force/torque (F/T) sensor mounted on a robotic arm. The AFP was brought into contact with a SUS 430 plate and subjected to a reciprocating sliding motion with a stroke length of 30 mm. During the test, the friction force along the sliding direction was continuously measured through the F/T sensor.

The reciprocating motion was repeated for 40,000 cycles. Figure 5b shows the friction force history during the repeated sliding test. Although the absolute magnitude of the friction force slightly decreased over the repeated cycles, the characteristic asymmetric friction pattern remained clearly observable throughout the experiment.

To quantitatively compare the anisotropic friction behavior before and after the durability test, the friction forces in the high-friction and low-friction directions were extracted from the initial and final cycle windows. The corresponding friction difference was defined as $\Delta F=F_{\mathrm{high}}-F_{\mathrm{low}}.$ As shown in Figure 5c, both directional friction forces decreased slightly after repeated sliding; however, the friction difference ΔF remained at a comparable level even after 40,000 cycles. This result indicates that the anisotropic friction mechanism generated by the selective cyanoacrylate coating was preserved during long-term cyclic operation.

#### 2.2.4. AFP Geometric Optimization

This section integrates two complementary analyses to determine the key geometric parameters that enhance the efficiency and stability of crawling locomotion. The first part evaluates how the inclination angle of the AFP influences the continuity and smoothness of ground contact during crawling. The second part examines how the curvature of the AFP affects its ability to conform to curved pipe surfaces, thereby improving adhesion stability in in-pipe environments.

**Figure 6:**
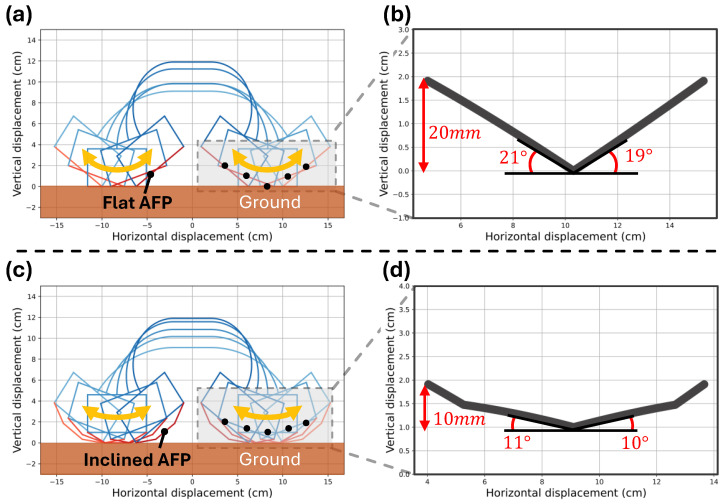
Constant-curvature crawling simulation with flat and inclined AFPs. (**a**) Crawling sequence with a flat AFP ($\theta_{a}=0^{{}^{\circ}}$). (**b**) Endpoint trajectory for $\theta_{a}=0^{{}^{\circ}}$. (**c**) Crawling sequence with an inclined AFP ($\theta_{a}=20^{{}^{\circ}}$). (**d**) Endpoint trajectory for $\theta_{a}=20^{{}^{\circ}}$.

To investigate how AFP inclination contributes to smooth crawling, a constant-curvature model was used as a simplified kinematic representation of the robot bending configuration under different geometric configurations of the AFP ($\theta_{a}$). Rather than reconstructing the detailed curvature distribution of the origami backbone, the model describes how the bending of the compliant backbone changes the relative orientation between the drive modules during crawling. The simulation results are presented in Figure 6 and were derived from the mathematical modeling described in Section 3.2, where the detailed formulation and modeling procedure are explained.

The robot’s crawling motion when $\theta_{a}=0^{{}^{\circ}}$ (flat AFP) and $\theta_{a}=20^{{}^{\circ}}$ (inclined AFP) is shown in Figure 6a and Figure 6c, respectively. In these figures, the blue regions schematically represent the control module, body frame, and drive module, illustrating how the robot deforms during crawling. Each figure superimposes five sequential configurations within one crawling cycle to visualize the periodic deformation process.

In the case of the flat AFP (Figure 6a), the robot primarily contacts the ground through the edge regions of the AFP, causing the contact point to shift abruptly between phases of the crawling cycle. This abrupt shift in the contact point leads to sharp and discontinuous changes in posture, as confirmed by the endpoint trajectory shown in Figure 6b. Figure 6b shows the trajectory of a representative point located at the end of the robot under this condition, where the vertical displacement reaches up to approximately 20 mm and the inclination angle varies sharply by up to $40^{{}^{\circ}}$. Such discontinuous transitions and large displacement variations indicate potential mechanical instability during the crawling process and can impose substantial load on the actuators, increasing the risk of slip and control errors.

In contrast, when the inclined AFP ($\theta_{a}=20^{{}^{\circ}}$) is applied, as shown in Figure 6c, contact with the ground is maintained more uniformly across the AFP surface, and the contact point shifts smoothly and continuously throughout the crawling cycle. Figure 6d presents the trajectory of the same endpoint, showing that the vertical displacement decreases to about 10 mm and the angular variation reduces to approximately $21^{{}^{\circ}}$. These results indicate that the inclined AFP maintains more continuous ground contact during bending, resulting in smoother posture transitions throughout the crawling cycle.

To further investigate the influence of AFP inclination on crawling stability, additional inclination angles were evaluated using the same constant-curvature simulation framework. Table 2 summarizes the resulting endpoint angular variation and vertical displacement for four representative inclination angles ($\theta_{a}=0^{{}^{\circ}}$, $10^{{}^{\circ}}$, $20^{{}^{\circ}}$, and $30^{{}^{\circ}}$).

As the inclination angle increases from $0^{{}^{\circ}}$ to $20^{{}^{\circ}}$, both the angular variation and the vertical displacement decrease significantly. This trend indicates that moderate AFP inclination improves the continuity of ground contact during crawling by preventing abrupt edge contact and distributing the contact region more evenly across the AFP surface.

When the inclination angle is further increased to $30^{{}^{\circ}}$, however, the improvement becomes marginal and the vertical displacement slightly increases again. This behavior suggests that excessive inclination reduces the effective contact region during certain phases of the crawling cycle, which may limit further improvement in locomotion stability.

Based on these results, an inclination angle of $\theta_{a}=20^{{}^{\circ}}$ was selected as the optimal configuration for the AFP. This angle provides the smallest vertical displacement and the most stable contact transition during crawling while avoiding excessive geometric inclination that may reduce effective contact area. Therefore, $\theta_{a}=20^{{}^{\circ}}$ was adopted in the final AFP design used in the experiments presented in this study.

**Figure 7:**
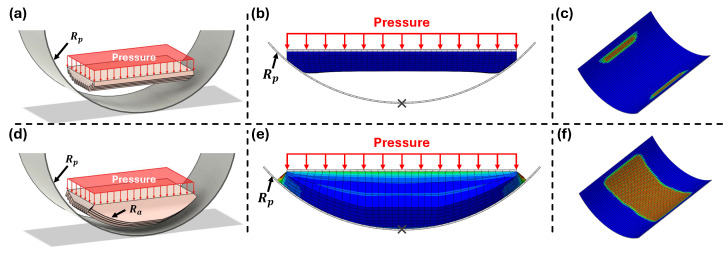
Finite element analysis of AFP contact on curved pipes. (**a**) Simulation setting for the flat AFP ($R_{a}=0$). (**b**) Deformation and stress distribution for $R_{a}=0$ under applied pressure. (**c**) Contact region for $R_{a}=0$ on the pipe surface. (**d**) Simulation setting for the curved AFP ($R_{a}=125$ mm). (**e**) Deformation and stress distribution for $R_{a}=125$ mm. (**f**) Contact region for $R_{a}=125$ mm on the pipe surface.

Following this analysis of AFP inclination, the adaptive adhesion behavior of the AFPs was examined to determine how well they conform to different pipe curvatures. Finite element analysis (FEA) was conducted to evaluate the normal-contact conformity of the AFP under varying curvature radii, and the results are shown in Figure 7.

The analysis was performed using Abaqus/Standard 2025 (Dassault Systèmes, Providence, RI, USA). The pipe was modeled as a rigid body, and the AFP was defined as a hyperelastic material using the Yeoh 3rd-order model for Ecoflex 00-10 ($C_{10}=0.00457754$, $C_{20}=0.000159533$, $C_{30}=-2.9721\times 10^{-7}$, $R^{2}=0.9984$) [42]. Surface-to-surface contact was applied with a hard contact condition along the normal direction and a zero-friction Penalty formulation along the tangential direction to isolate normal contact behavior. This simplified contact condition was intentionally used to evaluate the geometric conformity of the AFP to the pipe surface without coupling effects from tangential friction. By eliminating tangential constraints, the analysis focuses on how the AFP curvature influences the normal contact distribution and effective contact area. The bottom of the pipe was fully fixed, and a uniform pressure of 1 kPa was applied vertically to the AFP surface. This value was selected as a conservative contact loading condition, slightly higher than the normal loads used in the friction experiments, to account for additional normal forces that may arise during tendon-driven locomotion.

The simulation setups for the flat AFP ($R_{a}=0$) and curved AFP ($R_{a}=125$ mm) are shown in Figure 7a and Figure 7d, respectively. The corresponding deformation results under pressure, visualized with stress contours to show how each AFP conforms to the pipe surface, are presented in Figure 7b,e. In these results, the flat AFP exhibits limited conformity, whereas the curved AFP deforms more uniformly along the pipe curvature.

The resulting contact regions, isolated by hiding the AFP geometry, are shown in Figure 7c,f, allowing direct comparison of the actual pipe–AFP contact area. The flat AFP contacts only a narrow portion of the pipe surface, while the curved AFP achieves a continuous and substantially larger contact region.

The analysis was repeated for three pipe curvatures ($R_{p}=100$, 125, and 150 mm), and the results are summarized in Table 3. In all cases, the curved AFP ($R_{a}=125$ mm) consistently achieved a larger contact area than the flat AFP ($R_{a}=0$), demonstrating its enhanced ability to conform to the inner pipe surface. This adaptive contact behavior improves adhesion stability and frictional performance during locomotion, thereby supporting reliable in-pipe crawling.

#### 2.2.5. Effect of Embedded Magnet on Frictional Performance

The anisotropic friction behavior of the AFP is primarily generated by the ridge geometry and selective surface coating described in Section 2.2.1. In addition to these friction-generating mechanisms, a neodymium magnet was embedded inside the AFP to enhance adhesion when operating on metallic pipe surfaces. Because magnetic attraction can increase the effective normal contact force between the AFP and the surface, it is necessary to clarify whether the observed locomotion performance originates mainly from frictional anisotropy or from magnetic assistance.

To isolate the contribution of magnetic adhesion, an additional friction experiment was conducted by comparing AFP samples with and without embedded magnets. Both samples were fabricated using the optimized AFP geometry determined in Section 2.2.4, ensuring that the only difference between the two samples was the presence of the magnet. Friction forces were measured along both the high-friction and low-friction directions of the AFP.

**Figure 8:**
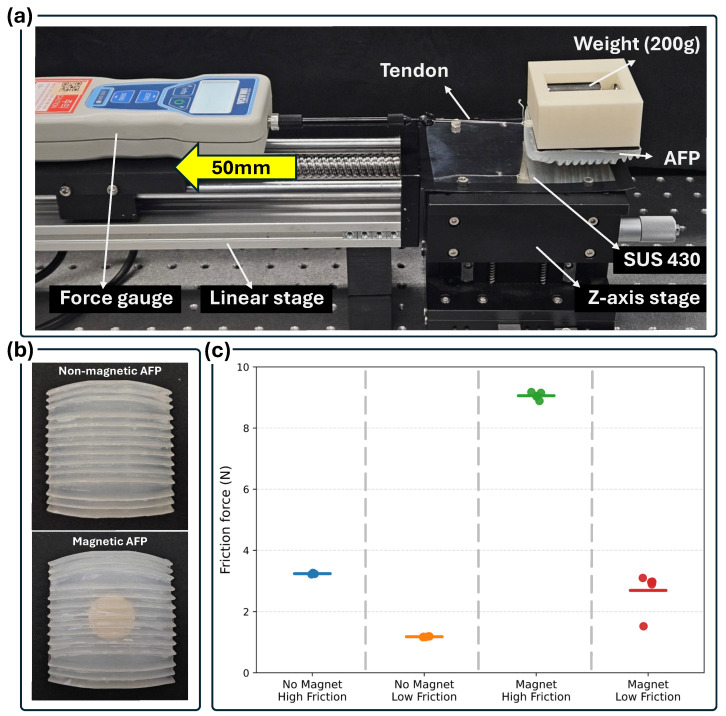
Experimental comparison of anisotropic friction behavior between magnetic and non-magnetic AFPs. (**a**) Friction test setup using a linear stage and force gauge with a SUS 430 plate under a constant normal load of 200 g. (**b**) Fabricated AFP samples without and with an embedded neodymium magnet. (**c**) Measured friction forces in the high-friction and low-friction directions for both configurations (five repeated trials). Dots represent individual measurements from repeated trials, and lines indicate the corresponding mean values.

The experimental setup is shown in Figure 8a. The AFP specimen was placed on a SUS 430 plate mounted on a linear stage, and a constant normal load of 200 g was applied vertically using a weight to reproduce the contact conditions during locomotion. The stage was then translated horizontally at a constant speed of 5 mm/s over a sliding distance of 50 mm while the friction force was measured using a force gauge. For each AFP configuration (magnetic and non-magnetic), the friction test was repeated five times for both the high-friction and low-friction directions.

The measured friction forces are summarized in Figure 8c. In both the magnetic and non-magnetic AFPs, a clear difference between the high-friction and low-friction directions was observed, confirming that the anisotropic friction behavior originates from the ridge geometry and selective surface coating. When the magnet was embedded, the absolute magnitude of the friction force increased significantly due to the additional normal force generated by magnetic attraction. In particular, the friction force in both the high-friction and low-friction directions increased by approximately 300% compared to the non-magnetic AFP. This increase occurred in both directions, indicating that the magnet uniformly amplifies the overall contact force rather than altering the directional friction mechanism. Importantly, despite the increase in magnitude, the ratio between the high-friction and low-friction directions remained similar for both magnetic and non-magnetic AFPs, indicating that the directional friction mechanism is independent of magnetic adhesion.

These results indicate that the embedded magnet primarily enhances adhesion by increasing the overall contact force between the AFP and the metallic surface. In contrast, the directional friction mechanism responsible for locomotion is governed by the anisotropic ridge structure and surface treatment. Therefore, the magnet functions as an auxiliary adhesion mechanism rather than the primary source of directional locomotion.

## 3. Locomotion and Control

### 3.1. Multi-Gait Crawling Modes

**Figure 9:**
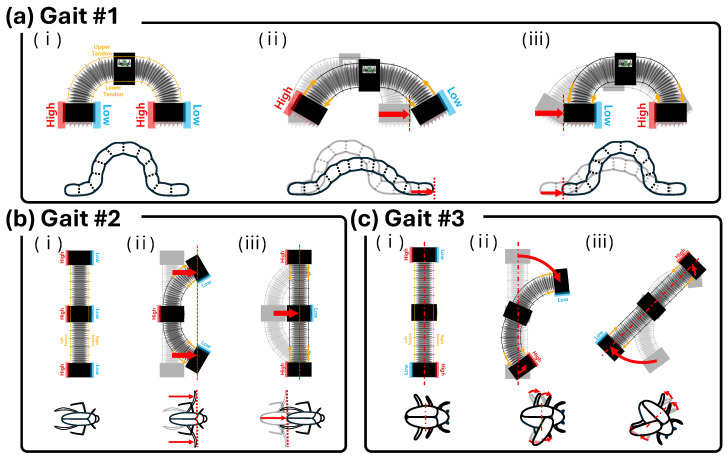
Bioinspired crawling gait modes of the proposed robot. (**a**) Longitudinal locomotion inspired by caterpillar (gait #1). (**b**) Transverse locomotion inspired by the water boatman (gait #2). (**c**) Rotational locomotion inspired by the whirligig beetle (gait #3). (i)–(iii) indicate the sequential deformation states within one locomotion cycle.

The three locomotion modes of the proposed robot, inspired by the movement principles of biological organisms, are illustrated in Figure 9: (a) longitudinal motion (gait #1), (b) transverse motion (gait #2), and (c) rotational motion (gait #3). Each mode is enabled by tendon-driven deformation combined with AFPs that possess directional friction, as described in Section 2.2. With their friction orientations assigned appropriately for each gait, the AFPs generate directional mobility and anchoring that collectively produce net locomotion.

The forward crawling mode inspired by caterpillar locomotion is shown in Figure 9a.

Caterpillars are representative soft-bodied crawlers that move through sequential interactions between different body segments and the ground. During locomotion, the posterior prolegs first attach firmly to the substrate and act as an anchor while the anterior body segments extend forward. In the subsequent phase, the anterior segments attach to the substrate while the posterior body contracts and is pulled forward. This alternating anchor–advance mechanism allows the caterpillar to generate net forward locomotion. The key principle underlying this motion is that different body segments alternately function as either an anchored support or a moving segment through asymmetric interaction with the environment.

A caterpillar advances by anchoring its rear segment while extending the front, and then anchoring the front while pulling the rear forward [4,43]. To reproduce this alternating anchoring–advancing mechanism, the bottom AFPs are oriented such that the forward direction corresponds to low friction and the backward direction corresponds to high friction.

During the first phase (Figure 9(a,ii)), the upper tendon contracts and the lower tendon relaxes, causing the body to expand outward. Because the front AFP faces the outward (forward) direction of motion, it experiences low friction and advances, while the rear AFP faces its outward (backward) direction, where friction is high, and therefore remains fixed.

During the second phase (Figure 9(a,iii)), the upper tendon relaxes and the lower tendon contracts, pulling the body inward. In this state, the inward direction for the rear AFP corresponds to its low-friction orientation, allowing the rear to advance, while the front AFP—now facing inward, where friction is high—anchors. Repeating this alternating sequence results in continuous forward crawling, and reversing the friction orientation enables backward locomotion.

The transverse locomotion mode inspired by the propulsion strategy of the water boatman is presented in Figure 9b.

Water boatmen swim on the water surface using their hind legs as paddles. Their propulsion relies on asymmetric hydrodynamic drag generated during the leg stroke cycle. When the legs move forward in a folded configuration, their projected area is small, resulting in low fluid resistance. In this phase, the legs move forward relative to the body while the body remains nearly stationary. In contrast, when the legs extend and sweep backward, their projected area increases significantly, generating large hydrodynamic drag. The legs therefore act as a temporary anchor against the water while the body moves forward relative to the legs. The propulsion of the insect thus emerges from the asymmetric drag between the forward and backward strokes.

The water boatman minimizes drag when folding its legs forward and maximizes drag when sweeping them backward, producing lateral thrust [44,45]. To reproduce this directional drag asymmetry, the side AFPs are oriented such that the right-facing direction corresponds to low friction and the left-facing direction corresponds to high friction for rightward locomotion.

During the first phase (Figure 9(b,ii)), the right tendon contracts and the left tendon relaxes, bending the body to the right. As the body bends, the front and rear modules shift rightward along the curvature and encounter the right-facing low-friction orientation, allowing them to slide. In contrast, the central module shifts leftward and faces the high-friction orientation, causing it to anchor.

During the second phase (Figure 9(b,iii)), the right tendon relaxes and the left tendon contracts, bending the body to the left. The central module now shifts rightward and encounters the low-friction orientation, enabling it to advance, while the front and rear modules shift leftward and experience high friction, remaining fixed.

By alternating the rightward motion of the end modules and the rightward motion of the central module across the two phases, the robot achieves continuous rightward translation. Reversing the friction orientation enables leftward locomotion.

The rotational locomotion mode inspired by the whirligig beetle is illustrated in Figure 9c.

Whirligig beetles are known for their rapid rotational maneuvers on the water’s surface. During turning, one of the elytra partially unfolds, increasing hydrodynamic drag on that side of the body. At the same time, the hind legs continue to generate propulsion. Because the drag differs between the two sides of the body, an asymmetric resistance distribution is produced, which generates a rotational moment. This asymmetric interaction with the surrounding fluid enables the beetle to rotate efficiently while maintaining propulsion.

When rotating on the water’s surface, the beetle unfolds only one of its elytra, increasing drag on that side while generating thrust with its hind legs, which produces a net rotational moment [46,47]. To emulate this asymmetric drag mechanism, the side AFPs are oriented such that the clockwise tangential direction corresponds to low friction and the counterclockwise direction corresponds to high friction.

During the first phase (Figure 9(c,ii)), the right tendon contracts and the left tendon relaxes, bending the body to the right. As the body bends, the front and rear modules shift rightward relative to the curvature. For the front module, the rightward direction matches its low-friction orientation, allowing it to slip rightward; combined with the bending geometry, this slip generates a clockwise rotation of the module. In contrast, the rear module encounters high friction in the rightward direction and therefore anchors.

During the second phase (Figure 9(c,iii)), the right tendon relaxes and the left tendon contracts, bending the body to the left. Both modules now shift leftward. In this configuration, the rear module’s leftward direction corresponds to its low-friction orientation, allowing it to slip and rotate clockwise, while the front module—facing high friction—remains fixed.

By alternating these two phases, the robot achieves continuous clockwise rotation, and reversing the friction orientation enables counterclockwise rotation.

### 3.2. Gait Transition Mechanism

**Figure 10:**
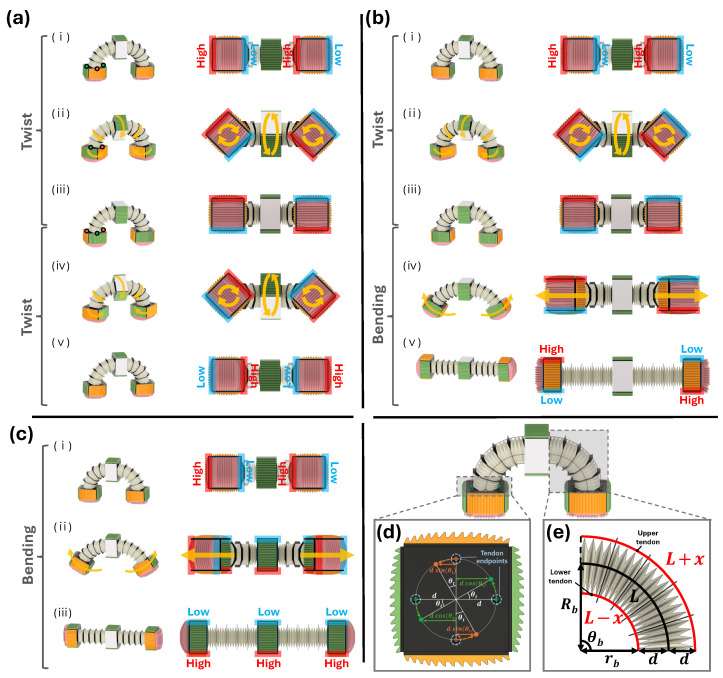
Gait-transition motions enabling different gait configurations. (**a**) Transition from forward to backward locomotion within gait #1. (**b**) Transition from gait #1 to gait #3. (**c**) Transition from gait #1 to gait #2. (**d**) Tendon-endpoint trajectories during twist. (**e**) Tendon-length relationships during bending. In (**a**–**c**), (i–v) indicate the sequential deformation states within one transition cycle.

An overview of the transition mechanism between the three gait modes is provided in Figure 10a–c. In each panel, the AFPs used for gait #1 (pink), gait #2 (green), and gait #3 (orange) are highlighted to show how the friction orientations change throughout the transition. The left panels illustrate the robot’s deformation during the gait transition, while the right panels present the corresponding bottom views.

Frames (i)–(iii) in Figure 10a represent the twisting motion. During twisting, the robot undergoes a torsional motion around its longitudinal axis, causing the front AFP to exhibit a counterclockwise relative motion and the rear AFP to exhibit a clockwise relative motion. During twisting, the robot must rotate while keeping the AFPs approximately parallel to the ground, because losing parallel contact would cause the AFPs to lift off, reducing friction, degrading performance, and compromising stability.

As shown in Figure 10d, each tendon endpoint is offset laterally from the backbone by a distance *d*. In this figure, the orange point denotes the left–right tendon endpoints, and the green point denotes the upper–lower tendon endpoints. Maintaining parallel ground contact requires each endpoint to follow a circular trajectory of radius *d* around the backbone during twisting. Because the endpoints follow circular trajectories during twisting, the effective tendon-length variation is determined by the projection of their motion onto the longitudinal axis of the robot. For the upper–lower tendon endpoints (green points), the projected displacement becomes $d-d\cos\theta_{t}$, whereas for the left–right tendon endpoints (orange points), the projected displacement becomes $d-d\sin\theta_{t}$. These projected displacements match the length variations produced by winding or unwinding at the pulleys.

Repeating this twisting motion twice yields a $180^{{}^{\circ}}$ rotation of the friction configuration, transitioning the robot from the gait #1 (forward-motion) orientation in state (i) to the backward-motion orientation in state (v).

The transition from gait #1 to gait #3, which requires a combined twist-and-bending sequence, is shown in Figure 10b. Here, “bending” refers to the deformation between a straight and a curved configuration produced by differential tendon actuation. After completing the twist phase (frames (i)–(iii)), the robot performs bending (frames (iii)–(v)), which spreads the drive modules apart and brings the side AFPs into contact with the ground.

The tendon-length relation arising from bending is depicted in Figure 10e. Let *L* denote the centerline arc length of the robot, $R_{b}$ the bending radius of the backbone, and $\theta_{b}$ the bending angle such that $L=R_{b}\theta_{b}$. In this robot, the backbone arc length *L* is fixed at 108 mm. With the same geometric offset *d* described earlier, the radius of the inner tendon path becomes $r_{b}=R_{b}-d$. Because the inner tendon travels a shorter path than the backbone, the tendon-length change *x* satisfies $L-x=r_{b}\theta_{b}$. Substituting $L=R_{b}\theta_{b}$ yields

$$x=d\theta_{b}.$$

Using the pulley radius $r_{p}$, the motor rotation needed to generate this tendon displacement satisfies

$$2\pi r_{p}n=2x.$$

Since the bending motion for gait #3 requires a rotation of $\Delta \theta_{b}=\pi /2$, the required number of motor turns follows directly from the above expression.

By executing the twist followed by bending, the robot transitions smoothly between gait #1 and gait #3. Reversing the twist direction produces the opposite orientation of gait #3.

The transition between gait #1 and gait #2 is illustrated in Figure 10c. In this case, only bending is required to obtain the gait #2 friction orientation; twisting is not needed. Alternatively, two consecutive twists followed by the same bending motion produce the opposite orientation of gait #2.

## 4. Results

### 4.1. Flat-Surface Evaluation

**Figure 11:**
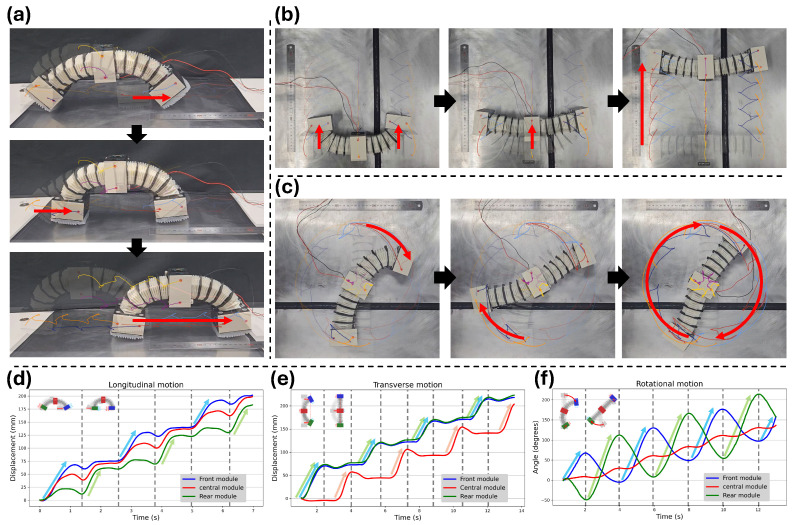
Motion capture results for the three locomotion modes on a flat SUS 430 surface. (**a**) Motion capture sequence of gait #1. (**b**) Motion capture sequence of gait #2. (**c**) Motion capture sequence of gait #3. (**d**) Measured forward displacement profile for gait #1. (**e**) Measured lateral displacement profile for gait #2. (**f**) Measured rotational displacement profile for gait #3. Colored arrows in (**d**–**f**) indicate the direction and relative magnitude of the dominant displacement of each module during a gait cycle.

The motion capture results of the three locomotion modes (gait #1–#3) of the proposed robot are presented in Figure 11. All experiments were conducted on a flat SUS 430 surface, and the robot’s motion was recorded using a camera-based tracking setup (Video S1). Two markers were attached to each module, and the centroid position of each module was used to compute the translational or rotational displacement throughout the motion. Each experiment quantitatively evaluates the postural deformation and displacement produced during longitudinal, transverse, and rotational locomotion.

The motion capture sequence of gait #1 (longitudinal locomotion) is shown in Figure 11a. The periodic expansion and contraction of the body are clearly visible, and the intended anchoring–advancing pattern emerges naturally: during the expanding phase, the front module advances while the rear module remains fixed, and during the contracting phase, the front module anchors while the rear module moves forward. In particular, the robot maintains continuous ground contact throughout the cycle, demonstrating the smooth crawling behavior targeted in the AFP design of Section 2.2.4. Through this visually consistent and uninterrupted motion pattern, the robot produces smooth and continuous forward locomotion.

The motion capture sequence of gait #2 (transverse locomotion) is depicted in Figure 11b. The lateral bending motion of the body is clearly expressed, and the alternation of motion between the side modules and the central module appears as designed. During the contracting phase, the side modules shift laterally while the central module remains fixed, and during the expanding phase, the central module moves while the side modules anchor. This visually repeatable deformation pattern results in stable and well-defined transverse locomotion.

The motion capture sequence of gait #3 (rotational locomotion) is illustrated in Figure 11c. The motion capture sequence clearly shows the alternating rightward and leftward bending that characterizes this gait. During the contracting phase, the body bends to the right, causing the front module to slip clockwise while the rear module anchors. During the expanding phase, the body bends to the left, causing the rear module to slip clockwise while the front module anchors. By visually confirming this alternating slip–anchor pattern, it is evident that the robot generates steady and cumulative clockwise rotation exactly as intended in the gait design.

The lower plots in Figure 11d–f provide the quantitative motion capture data for each locomotion mode, expressed as time-dependent linear displacement or angular rotation. The blue, red, and green traces represent the trajectories of the front module, central module, and rear module, respectively.

The longitudinal locomotion result (gait #1) is shown in Figure 11d. The front and rear modules exhibit clear out-of-phase displacement patterns, in which one module advances while the other remains nearly stationary. This alternating motion produces a distinct anchoring–sliding sequence, and the plateau regions in each trajectory indicate that back-slip during anchoring is negligible. The peak-to-peak displacement amplitudes of the two modules remain consistent across cycles, confirming stable actuation and repeatable mechanical deformation. Each cycle generates approximately 70 mm of net forward motion and requires about 2.5 s, resulting in a total displacement of nearly 200 mm over 7 s.

The transverse locomotion result (gait #2) is presented in Figure 11e. The front–center– rear trajectories form a highly repeatable lateral displacement pattern, where the side modules (front and rear) move simultaneously during the contracting phase, followed by the central module moving during the expanding phase. Because this side–center exchange occurs with nearly identical displacement amplitudes in each phase, the rightward and leftward motions appear symmetric in the time history, demonstrating that transverse locomotion does not rely on asymmetric slip. Each cycle produces roughly 55 mm of net lateral translation over approximately 3.5 s, yielding a cumulative displacement of around 220 mm in 14 s.

The rotational locomotion result (gait #3) is illustrated in Figure 11f. The front and rear modules exhibit alternating angular motions: during each bending phase, the module oriented toward the low-friction direction rotates with a larger amplitude, while the opposing module undergoes only a smaller rotation due to its high-friction orientation. This unequal but coordinated behavior repeats consistently across cycles and reflects the intended slip–anchor mechanism of the rotational gait. As a result of this alternating pattern, the central module shows a smooth and steadily increasing angular displacement, representing the accumulated net rotation of the entire body. In other words, although the end modules alternate between active rotation and partial anchoring, their combined effect generates a stable and unidirectional rotational progression. Each rotational cycle produces approximately $50^{{}^{\circ}}$ of clockwise rotation and takes about 4 s, resulting in a total rotation of roughly $150^{{}^{\circ}}$ over 13 s.

Across all modes, the corresponding average speeds were approximately 28.6 mm/s for forward crawling, 15.7 mm/s for transverse locomotion, and 11.5°/s for rotational motion.

To further benchmark the proposed robot against existing in-pipe robotic systems, a quantitative comparison with representative soft in-pipe robots is summarized in Table 4. The comparison includes actuator count, locomotion type, and reported locomotion speed, which are commonly used metrics for evaluating in-pipe robotic performance. Most previously reported soft in-pipe robots primarily achieve axial crawling using pneumatic, buckling-based, or single-actuator mechanisms. In contrast, the proposed robot achieves axial forward/backward crawling, transverse translation, and in-place rotation using only two actuators. Compared with representative soft in-pipe robots reported in the literature, including the single-actuator crawler by Lin et al. (2023) [39], the buckling-based multi-locomotion robot developed by Yeh et al. (2020) [34], the untethered soft in-pipe robot by Zhao et al. (2024) [41], and the annelid-inspired peristaltic soft robot by Martinez et al. (2023) [48], the proposed system provides a broader locomotion repertoire while maintaining competitive axial crawling speed.

### 4.2. Validation Under Varying Pipe and Surface Conditions

To further evaluate the applicability of the proposed robot in more realistic in-pipe environments, additional locomotion experiments were conducted under varying pipe geometries, inclined orientations, and different surface conditions. These experiments were designed to examine how changes in curvature, gravity-induced loading, and interfacial lubrication affect the locomotion performance of the anisotropic friction-based robot.

During the experiments, visual tracking markers were attached to the front, central, and rear modules of the robot. Two markers were placed on each module, and their trajectories were extracted from the recorded video frames to quantify the locomotion behavior. The displacement histories presented in the following graphs correspond to the average x-position of the two tracked nodes on the central module, representing the overall translational motion of the robot during crawling.

**Figure 12:**
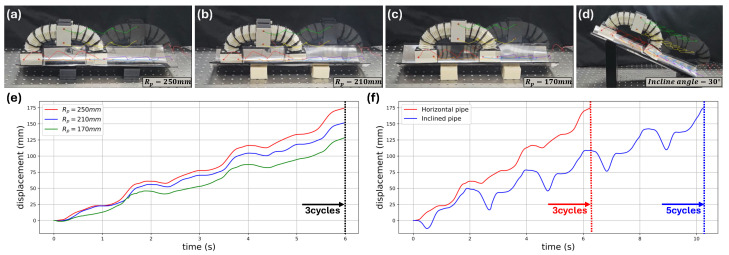
Validation of locomotion under varying pipe geometries and inclined orientation. (**a**–**c**) Crawling experiments in pipes with curvature radii of 250 mm, 210 mm, and 170 mm, respectively. The colored curves represent the trajectories of tracked nodes attached to the front, central, and rear modules of the robot. (**d**) Crawling experiment in an inclined pipe with a 30° orientation. (**e**) Displacement histories under different pipe curvature conditions, calculated from the average x-position of the two tracked nodes on the central module. (**f**) Comparison of displacement histories between horizontal and inclined pipe conditions.

First, locomotion experiments were performed in pipe environments with different curvature radii, as shown in Figure 12a–c. The plotted curves represent the trajectories of the tracked nodes during crawling. The robot was tested in three pipe conditions with curvature radii of 250 mm, 210 mm, and 170 mm, respectively. For all three conditions, three crawling cycles were executed within approximately 6 s. The resulting central-module displacements were approximately 175 mm, 150 mm, and 130 mm, respectively, as shown in Figure 12e.

As the pipe curvature increased, the locomotion displacement gradually decreased. This reduction is attributed to the increased geometric mismatch between the AFP curvature and the inner pipe surface, which reduces the effective contact condition during crawling. Nevertheless, stable longitudinal locomotion was maintained in all three cases. This result supports the passive adaptability of the AFP design to different pipe curvatures and is consistent with the finite element analysis in Section 2.2.4, where the curved AFP was shown to maintain distributed contact over a range of pipe geometries.

To further assess locomotion robustness under gravity-driven loading, an additional experiment was conducted in an inclined pipe with a curvature radius of 250 mm and an inclination angle of 30°, as shown in Figure 12d. The comparison between the horizontal and inclined pipe conditions is presented in Figure 12f. In the horizontal pipe, the robot reached approximately 175 mm within three crawling cycles, whereas in the inclined pipe, the robot required five cycles and about 10 s to reach a comparable displacement.

The slower locomotion in the inclined condition is attributed to intermittent slip induced by the additional gravitational load acting along the pipe direction. Even under this condition, however, the robot maintained repeated forward locomotion without complete loss of contact or locomotion failure. This result indicates that the proposed robot preserves its basic crawling functionality under inclined pipe conditions, although slip suppression under gravity-assisted loading should be further improved in future designs.

**Figure 13:**
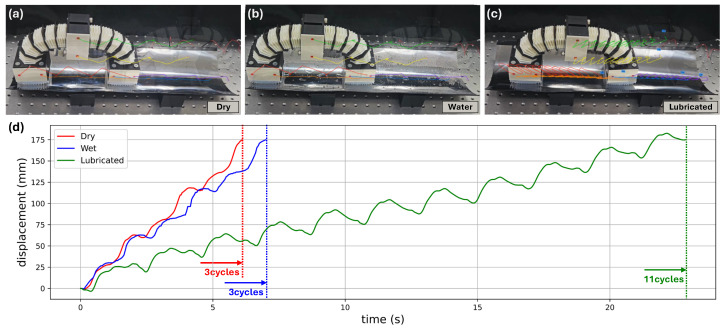
Validation of locomotion under different surface conditions. (**a**) Crawling on a dry SUS 430 surface. (**b**) Crawling on a water-covered SUS 430 surface. (**c**) Crawling on an oil-lubricated SUS 430 surface. (**d**) Displacement histories under the three surface conditions, obtained from the average x-position of the two tracked nodes on the central module.

Next, the influence of surface condition on locomotion was investigated using three different SUS 430 interface states: a dry surface, a water-covered surface, and an oil-lubricated surface, as shown in Figure 13a–c. The corresponding displacement histories are presented in Figure 13d.

On the dry surface, the robot reached approximately 175 mm within three crawling cycles. Under the water-covered condition, the locomotion performance was slightly reduced compared with the dry condition, but the overall displacement trend remained similar and the robot still achieved nearly the same travel distance within a comparable number of cycles. This result suggests that the anisotropic friction mechanism is relatively robust to moderate moisture on the pipe surface.

In contrast, the oil-lubricated surface caused a substantial deterioration in locomotion performance. In this condition, the robot required approximately 11 crawling cycles and about 22 s to travel the same distance of 175 mm. Because the locomotion mechanism of the proposed robot relies on directional friction asymmetry, the presence of lubricant significantly reduces the friction difference between the high-friction and low-friction directions of the AFP, thereby weakening the anchor–slip effect responsible for net forward motion.

Overall, the additional experiments demonstrate that the proposed robot maintains functional locomotion over a range of pipe curvatures, under inclined loading, and on wet metallic surfaces. At the same time, the results clarify an important limitation of the current anisotropic friction strategy in lubricated environments, where the directional friction contrast becomes insufficient for efficient anchor–slip locomotion. These findings provide a more realistic assessment of the operating envelope of the proposed robot and complement the geometry-based design analysis presented earlier in the manuscript.

### 4.3. In-Pipe Demonstrations

To evaluate the robot’s ability to perform multi-gait locomotion under realistic in-pipe conditions, a custom pipe environment was constructed using SUS 430 stainless-steel sheets, a ferromagnetic material commonly used in industrial pipelines. Commercial metallic pipes are fully enclosed, making direct observation of internal motion difficult. Therefore, open-top cylindrical and T-junction structures were fabricated with a curvature radius of 250 mm, allowing visual access while preserving the magnetic adhesion and geometric characteristics of actual pipes.

**Figure 14:**
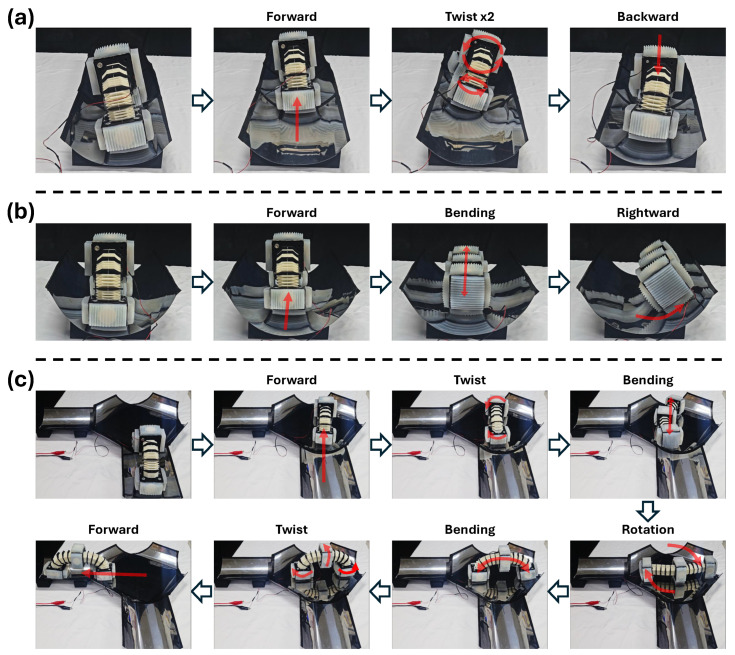
Demonstration of multi-gait locomotion inside the fabricated SUS 430 pipe environment. (**a**) Longitudinal locomotion: forward crawling, a twist-based gait transition, and backward return. (**b**) Longitudinal-to-transverse locomotion: forward crawling, a bending transition, and rightward motion inside the pipe. (**c**) Longitudinal-to-rotational locomotion in a T-junction: forward crawling, a twist–bending transition, clockwise rotation for heading adjustment, and continued forward movement along the selected path.

Three representative demonstrations of longitudinal, transverse, and rotational locomotion inside the fabricated pipe are summarized in Figure 14. All forward, rightward, and clockwise directions in this section follow the same top-down coordinate convention used throughout the paper (Video S2).

The longitudinal locomotion test is shown in Figure 14a. The robot first operates in gait #1 and crawls forward along the curved pipe wall. After reaching the end of the pipe, it performs two sequential twist transitions, switching the AFP orientation from the forward-locomotion configuration to the backward-locomotion configuration. Using this reversed friction alignment, the robot crawls backward along the same path and returns to its starting position while maintaining stable adhesion throughout the motion.

The combined longitudinal and transverse locomotion is presented in Figure 14b. The robot begins with gait #1 and moves forward inside the cylindrical pipe. It then performs the bending-based transition required to enter gait #2. After the transition, the side AFPs make contact with the pipe wall, allowing the robot to shift laterally. As a result, the robot generates a clear rightward transverse motion, demonstrating that the intended lateral gait is preserved even under the curved and confined geometry.

The rotational locomotion inside a T-shaped pipe junction is demonstrated in Figure 14c. The robot first approaches the junction in gait #1, then performs the twist–bending transition necessary to establish the gait #3 AFP configuration. Once the rotational mode is formed, the robot rotates clockwise in place until the desired heading angle is reached. After completing the rotation, the robot executes the reverse twisting–bending transition to return to the gait #1 configuration. With the forward-locomotion pattern recovered, the robot then proceeds to crawl reliably into the selected branch of the T-junction.

In addition to the qualitative demonstration of the gait transitions, the temporal characteristics of the transition process were also examined during the experiments. The twisting motion used to reorient the AFP configuration required approximately 3 s, while the subsequent bending motion used to establish side-AFP contact required approximately 1 s. During these transition stages, small positional drift was occasionally observed due to partial slip between the AFPs and the pipe surface. This slip mainly occurred because the pipe curvature slightly altered the local contact condition of the AFPs during twisting and bending deformation, resulting in minor position variations while the robot was reorienting its friction configuration. However, the magnitude of this drift remained relatively small and did not disrupt the intended locomotion sequence. After each transition, the robot consistently established the required friction configuration and successfully executed the target gait.

These experiments confirm that the proposed tendon-driven robot can reliably perform multi-gait locomotion—forward/backward crawling, lateral translation, and in-place rotation—within confined pipe environments. The successful demonstrations further validate the effectiveness of the AFPs and the robustness of the proposed gait-transition strategy under realistic geometric constraints.

### 4.4. Energy Consumption Analysis of Locomotion Modes

To quantitatively evaluate the energy consumption characteristics of the proposed locomotion mechanisms, the electrical power consumption of the actuators was measured during repeated locomotion cycles. Motor voltage and current were recorded during operation, and the instantaneous electrical power was calculated to analyze the temporal power consumption of each locomotion mode.

**Figure 15:**
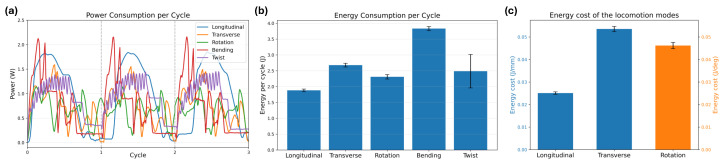
Energy consumption analysis of the proposed locomotion modes. (**a**) Instantaneous electrical power consumption during repeated locomotion cycles. (**b**) Energy consumption per motion cycle obtained by integrating the power profiles. (**c**) Energy cost of the locomotion modes (J/mm for longitudinal and transverse; J/deg for rotational).

Figure 15a shows the measured power profiles for different deformation and locomotion modes. Each motion exhibits a distinct power pattern depending on the magnitude of body deformation required during the actuation process. Among the analyzed motions, the bending motion exhibits the highest power peaks because it involves the largest structural deformation of the robot body. During bending, the body transitions from a flattened configuration to a significantly curved configuration (and vice versa), which requires substantial mechanical work.

Longitudinal locomotion shows the second highest peak power. This is primarily attributed to the large contraction of the origami-based body structure during the locomotion cycle. Because the origami body stores elastic energy, the actuators must overcome the elastic restoring force during contraction, resulting in relatively high instantaneous power demand.

In contrast, transverse and rotational locomotion exhibit lower peak power because the deformation from the nominal configuration is relatively smaller at each instant. Consequently, the instantaneous actuation demand during these motions remains relatively moderate.

The twist motion exhibits relatively large signal variation. This is because two motors operate simultaneously during the twisting motion and the measured power was obtained by summing the power of both actuators. Consequently, the observed fluctuations are likely influenced by measurement noise rather than purely reflecting mechanical load variations.

The energy consumption per cycle was obtained by integrating the power profiles over each motion cycle, and the results are summarized in Figure 15b. Longitudinal locomotion shows the lowest energy consumption per cycle because the total deformation required per cycle is relatively small and the motion duration is shorter than those of the other locomotion modes. In contrast, transverse and rotational locomotion require larger overall deformation to produce lateral translation and angular rotation, respectively, resulting in higher energy consumption per cycle.

Among all analyzed motions, bending shows the highest energy consumption per cycle because it requires the largest structural deformation and has the longest actuation duration. The relatively large variance observed in the twist energy results is consistent with the previously discussed measurement noise arising from the combined power measurement of two motors.

To compare the energy consumption demand of each gait, the energy cost was defined as the energy required to generate unit displacement. For longitudinal and transverse locomotion, the energy cost was calculated in J/mm, whereas for rotational locomotion it was calculated in J/deg. The resulting values are shown in Figure 15c. Longitudinal locomotion exhibits the lowest energy cost because it combines the smallest energy consumption per cycle with sufficiently large net forward displacement. In contrast, transverse locomotion shows a higher energy cost because larger body deformation is required to generate lateral translation, while the net displacement produced per cycle remains relatively limited compared with the deformation effort. Rotational locomotion also exhibits a relatively high energy cost, since repeated bending-based slip–anchor motions are required to generate angular displacement. These results indicate that longitudinal locomotion is the most energetically economical gait among the tested modes, whereas transverse and rotational locomotion demand greater energy relative to the output motion produced in each cycle.

Finally, the actuator margin was examined to estimate the feasibility of additional payload. The robot body weighs approximately 200 g excluding the AFP modules. The maximum measured power during the bending motion was approximately 2.1 W. Considering the operating voltage of 5 V, this corresponds to a peak current of approximately 0.42 A. The XC330-M288-T actuator used in this study has a stall current of approximately 1.8 A and a stall torque of 0.93 N·m. Using a linear current–torque approximation, the measured peak current corresponds to an estimated torque of approximately 0.22 N·m, which is about 23% of the stall torque.

These results indicate that the actuator does not operate near saturation even during the most deformation-intensive motion. Therefore, the proposed platform is expected to support additional light payload such as compact inspection sensors or miniature camera modules.These results suggest that lightweight payload integration may be feasible without approaching actuator saturation, although direct added-payload experiments are still required. However, the maximum payload capacity should be validated experimentally in future work.

This energy consumption analysis provides quantitative evidence that the proposed robot achieves multi-directional locomotion using minimal actuation while maintaining sufficient actuator margin.

## 5. Discussion

This study demonstrates that the integration of tendon-driven continuum deformation and anisotropic friction pads (AFPs) enables multi-directional locomotion within confined pipe environments. Unlike many conventional in-pipe robots that rely primarily on axial crawling or require multiple actuators and mechanical switching mechanisms, the proposed system achieves forward/backward crawling, transverse translation, and in-place rotation using only two motors and modular AFP interfaces.

The key locomotion principle arises from the interaction between body deformation and friction-direction programming at the contact interface. Tendon-driven bending and twisting deformation reorients the effective friction direction of the AFPs, enabling different anchor–slip sequences for distinct locomotion modes. This design separates the actuation mechanism from the frictional function, allowing gait transitions to be achieved through geometric reconfiguration rather than through additional mechanical switching components. Through this mechanism, the robot can realize three locomotion strategies inspired by biological systems: caterpillar-like crawling for axial motion, water-boatman-inspired propulsion for lateral translation, and whirligig-beetle-like asymmetric interaction for in-place rotation. In particular, this rotational motion arises from asymmetric slip–anchor interaction between the AFPs and the pipe surface, which generates a friction-induced torque that drives rotation of the entire body. Because the deformation speed is relatively low and the robot structure is approximately symmetric, the locomotion primarily operates in a friction-dominated regime where inertial effects remain negligible.

Although the robot does not aim to replicate the detailed biomechanics of the referenced organisms, the locomotion strategies share a common physical principle observed in biological systems: net motion emerges from asymmetric environmental interaction, such as alternating anchoring and slipping or asymmetric drag generation.

The constant-curvature representation was adopted as a simplified kinematic model to analyze posture transitions during crawling. This model allows evaluation of how the relative orientation of the drive modules evolves during bending and how this orientation change influences the contact behavior of the AFPs. In practice, however, the tendon-driven origami backbone may exhibit non-uniform curvature due to fold stiffness variation, tendon force redistribution, and environmental contact interactions. Consequently, the constant-curvature model should be interpreted as a first-order approximation that captures the overall deformation tendency of the robot rather than providing an exact prediction of the detailed backbone shape. For more accurate motion prediction and control in complex pipe environments, future implementations may incorporate sensor feedback—such as internal state sensing based on tendon displacement or external sensing modules—and integrate such information with model-based or data-driven feedback control strategies to compensate for deviations from the idealized curvature assumption.

The design of the AFP interface plays a critical role in ensuring reliable locomotion. The inclination angle of the bottom AFP was shown to reduce abrupt variations in ground contact during crawling, thereby improving the stability of longitudinal locomotion. The curvature radius of the AFP further enhances surface conformity on cylindrical pipe walls, increasing the effective contact area and improving anchoring stability.

The finite element analysis of AFP conformity was conducted with zero tangential friction in order to isolate the effect of geometry on normal contact distribution. While real pipe–AFP interactions involve frictional coupling that influences local sticking and sliding behavior, the present analysis intentionally removes tangential friction to evaluate the geometric contribution to surface conformity. Even under friction-coupled conditions, a curvature-matched AFP is expected to maintain a more distributed contact pattern than a flat interface, suggesting that the qualitative trend of improved conformity remains valid.

Friction experiments further indicate that the anisotropic friction behavior primarily originates from the ridge geometry and selective surface coating of the AFP. The embedded neodymium magnet mainly contributes to increasing the effective normal contact force, thereby improving adhesion on metallic surfaces rather than directly generating directional friction. Durability tests showed that the directional friction contrast remained clearly observable after 40,000 reciprocating sliding cycles, suggesting that the friction interface maintains its functional role under repeated locomotion. In addition, the origami backbone fabricated from NinjaFlex TPU did not exhibit observable structural damage during the repeated bending and twisting cycles used in the present locomotion experiments, although a dedicated fatigue-life study of the full backbone remains an important topic for future work.

Experimental validations on flat SUS 430 surfaces and in fabricated pipe environments demonstrate the effectiveness of the proposed locomotion mechanism. Motion capture analysis confirmed repeatable anchor–slip sequences for longitudinal, transverse, and rotational gaits. The robot achieved locomotion speeds of 28.6 mm/s in forward crawling, 15.7 mm/s in transverse motion, and 11.5°/s in rotational locomotion.

Additional experiments conducted in inclined pipe configurations showed that forward crawling can be maintained under gravity-induced loading, although intermittent slipping becomes more noticeable compared with horizontal conditions. Because the propulsion mechanism relies on directional friction asymmetry at the contact interface, environmental surface conditions may influence locomotion performance. While the additional experiments demonstrated that locomotion is preserved on water-wetted metallic surfaces, more systematic investigation under controlled humidity, lubrication, and contaminated surface conditions remains an important topic for future study.

Beyond locomotion, the proposed architecture provides a promising platform for pipeline inspection tasks. The compliant body structure enables stable wall contact, while the ability to perform both axial translation and in-place rotation allows circumferential scanning of pipe interiors. This capability makes the system suitable for integration of inspection modules such as miniature cameras, ultrasonic probes, eddy-current sensors, or gas-leak detectors.

The energetic analysis indicates that the locomotion strategy requires relatively modest actuation effort. The peak torque observed during bending corresponds to approximately 23% of the actuator stall torque, suggesting that the actuators operate well below their mechanical limits. This remaining actuation margin indicates that additional payloads, such as lightweight sensing modules, could be integrated without approaching actuator capacity. At present, the robot operates using position-controlled tendon actuation based on the onboard PID controller of the motors and relies on an external wired power supply during experiments.

Finally, the scalability of the system with respect to pipe diameter should be considered. The AFP curvature design improves conformity across different pipe radii, enabling stable contact in pipes with curvature radii of approximately 150 mm and larger. Extending the system to smaller pipe diameters would require reducing the overall body size and actuation system, which is currently constrained by motor dimensions and torque capability.

Another important consideration for practical pipeline deployment is locomotion in steeply inclined pipe segments. Although the proposed robot was able to maintain crawling motion inside a pipe inclined at 30°, intermittent slip was observed due to the increased gravitational component acting along the pipe axis. This observation suggests that the current adhesion strategy, which primarily relies on anisotropic friction combined with passive magnetic adhesion, may become less effective as the pipe inclination increases. To further improve locomotion robustness in steep or vertical pipelines, additional adhesion mechanisms could be incorporated in future designs. For example, suction-based adhesion mechanisms widely used in wall-climbing robots can generate controllable normal forces independent of surface friction, while gecko-inspired dry adhesive structures based on microfibrillar surfaces may provide strong shear adhesion while maintaining compliance with curved pipe surfaces. Integrating such adhesion-enhancement strategies with the current anisotropic friction framework could significantly extend the operational envelope of the robot, enabling reliable locomotion in more challenging pipeline environments.

Although the gait transition mechanism was successfully demonstrated in the experiments, minor positional drift was occasionally observed due to partial slip during the twisting and bending stages. This behavior mainly arises from variations in the local contact conditions between the AFPs and the pipe surface, particularly when the robot operates inside curved pipe segments. While the observed drift did not interfere with the successful execution of locomotion modes, improving transition accuracy will be beneficial for precise navigation in complex pipeline environments. In future implementations, integrating sensing modules together with onboard power sources could enable adaptive locomotion control and untethered operation in practical pipeline environments. For example, IMU sensors could monitor body orientation and stabilize posture when the robot operates in inclined or curved pipes, while thin pressure or contact sensors embedded near the AFP interfaces could detect anchoring conditions and slip events during gait transitions. Because the AFP contact interface requires minimal thickness to preserve surface conformity, ultra-thin flexible pressure sensors are particularly suitable for this purpose [49]. In addition, vision sensors could support environment perception and path planning inside complex pipe networks, allowing the robot to determine appropriate locomotion modes and plan transitions among longitudinal, transverse, and rotational motions. Such multi-modal sensory feedback could significantly improve locomotion robustness under varying pipe curvature, inclination, and surface conditions commonly encountered in practical pipeline environments.

## 6. Conclusions

This study presented a tendon-driven soft in-pipe robot capable of multi-directional locomotion by combining continuum deformation with programmable anisotropic friction interfaces. Through coordinated bending and twisting of the origami backbone, the robot reorients the effective friction direction of the AFPs and generates distinct anchor–slip locomotion patterns.

The AFP design was optimized through friction experiments, simplified kinematic modeling, and finite element analysis to improve surface conformity and directional friction performance. Experimental validation confirmed stable locomotion on flat metallic surfaces and in fabricated pipe environments, achieving speeds of 28.6 mm/s for longitudinal crawling, 15.7 mm/s for transverse translation, and 11.5°/s for rotational motion.

These results demonstrate that complex multi-gait locomotion in confined pipe environments can be achieved using minimal actuation by exploiting the interaction between continuum deformation and directional friction. The proposed architecture provides a compact and adaptable locomotion platform for confined-space navigation.

Future work will focus on improving locomotion robustness under highly lubricated or contaminated pipe surfaces, integrating onboard sensing for adaptive locomotion control, and developing untethered implementations for practical pipeline inspection applications.

## Figures and Tables

**Table 1:** Conceptual comparison between representative soft crawling and in-pipe robots and the proposed system.

System	Actuators	Motion Repertoire	Friction-Control Mechanism
Kim et al. (2023) [40]	1	Axial crawling	Curvature-modulated anisotropic friction
Lin et al. (2023) [39]	1	Axial crawling	Passive anisotropic friction
Yeh et al. (2020) [34]	2	Hop + axial forward/backward crawling	Buckling-mediated contact asymmetry
Zhao et al. (2024) [41]	Multiple	Axial crawling	Radial anchoring feet
This work	2	Axial forward/backward crawling/ transverse translation/in-place rotation	Programmable friction reorientation

**Table 2:** Quantitative comparison of AFP inclination angles based on constant-curvature simulation.

Inclination Angle ($\theta_{a}$)	Angular Var. (Front)	Angular Var. (Rear)	Vertical Disp.
$0^{{}^{\circ}}$	$21^{{}^{\circ}}$	$19^{{}^{\circ}}$	20 mm
$10^{{}^{\circ}}$	$15^{{}^{\circ}}$	$13.6^{{}^{\circ}}$	12 mm
$20^{{}^{\circ}}$	$11^{{}^{\circ}}$	$10^{{}^{\circ}}$	10 mm
$30^{{}^{\circ}}$	$11.5^{{}^{\circ}}$	$10^{{}^{\circ}}$	12 mm

**Table 3:** Contact areas of the flat and curved AFPs under different pipe curvature radii ($R_{p}$).

$R_{p}$	100 mm	125 mm	150 mm
flat AFP	$636.0\;{\mathrm{mm}}^{2}$	$817.4\;{\mathrm{mm}}^{2}$	$926.2\;{\mathrm{mm}}^{2}$
curved AFP	$4678.0\;{\mathrm{mm}}^{2}$	$3233.7\;{\mathrm{mm}}^{2}$	$4285.4\;{\mathrm{mm}}^{2}$

**Table 4:** Quantitative comparison of locomotion performance with representative in-pipe robots.

System	Actuators	Locomotion Type	Speed	Remarks
Lin et al. (2023) [39]	1	Axial crawling	up to 27 mm/s	Single-actuator soft in-pipe crawler
Yeh et al. (2020) [34]	2	Hop + axial forward/backward crawling	2.2 mm/s (crawling)	Buckling-based multi-locomotion robot
Zhao et al. (2024) [41]	Multiple	Axial crawling	1.27 mm/s	Untethered soft in-pipe robot
Martinez et al. (2023) [48]	1	Peristaltic crawling	∼4 mm/s	Annelid-inspired soft robot
This work	**2**	Axial crawling/transverse translation/in-place rotation	28.6 mm/s (axial)/ 15.7 mm/s (transverse)/ 11.5°/s (rotational)	Programmable friction reorientation

## Data Availability

Data will be made available on request.

## Supplementary Materials

The following supporting information can be downloaded at https://www.mdpi.com/article/10.3390/biomimetics11040285/s1: Video S1: Multi-gait locomotion on flat surfaces; Video S2: Adaptive multi-gait transitions in complex pipe geometries.
